# What Is New about the Semimembranosus Distal Tendon? Ultrasound, Anatomical, and Histological Study with Clinical and Therapeutic Application

**DOI:** 10.3390/life14050631

**Published:** 2024-05-15

**Authors:** Pere Iglesias-Chamorro, Albert Pérez-Bellmunt, Sara Ortiz-Miguel, Ingrid Möller, Juan Blasi, Juan Ortiz-Sagristà, Carlo Martinoli, Xavier Sanjuan, Maribel Miguel-Pérez

**Affiliations:** 1 Unit of Human Anatomy and Embryology, Department of Pathology and Experimental Therapeutics, Faculty of Medicine and Health Sciences (Bellvitge Campus), University of Barcelona, L’Hospitalet de Llobregat, 08907 Barcelona, Spain; centretars@centretars.com (P.I.-C.); ingridmoller@ub.edu (I.M.); 2 Basic Sciences Department, Universitat Internacional de Catalunya, Sant Cugat del Vallès, 08190 Barcelona, Spain; aperez@uic.cat (A.P.-B.); sara.mortiz@ub.edu (S.O.-M.); 3 ACTIUM Functional Anatomy Group, Sant Cugat del Vallés, 08190 Barcelona, Spain; 4 Unit of Human Anatomy and Embryology, Department of Surgery and Medical-Surgical Specialities, Faculty of Medicine and Health Sciences (Clinic Campus), University of Barcelona, 08036 Barcelona, Spain; 5 Unity of Histology, Department of Pathology and Experimental Therapeutics, Faculty of Medicine and Health Sciences (Bellvitge Campus), University of Barcelona, 08907 Barcelona, Spain; blasi@ub.edu; 6 Anesthesia Department, Fundació Puigvert, 08025 Barcelona, Spain; siometge@gmail.com; 7 Department of Health Sciences, Università di Genova, Via Antonio Pastore 1, 16132 Genova, Italy; carlo.martinoli@unige.it; 8 Unit of Pathological Anatomy, Department of Pathology and Experimental Therapeutics, Faculty of Medicine and Health Sciences (Bellvitge Campus), University of Barcelona, 08907 Barcelona, Spain; xsanjuang@ub.edu

**Keywords:** semimembranosus tendon division, ultrasound, anatomical and histological study, rotation, knee joint, meniscus

## Abstract

The semimembranosus muscle inserts into several tendons that are associated with some pathologies. Although ultrasound is useful for studying, diagnosing, and managing these pathologies, the correct interpretation of any images requires a clear knowledge of the related anatomical structures and the inter-related functions. We studied 38 cryopreserved non-paired knees from adult anatomical specimens and 4 non-paired knees from 29 to 38-week-old fetuses. The semimembranosus muscle and its tendons were located, observed, and injected under ultrasound guidance. The macroscopic anatomy was studied using dissection and anatomical cuts and the tendons were analyzed histologically. Measurements of muscle were taken 10 cm from the medial epicondyle and just before the tendon divided. The ultrasound facilitated the identification of the different divisions of the tendon of semimembranosus muscle and the rotation of the muscle and tendon from medial to posterior. An anatomical study confirmed this rotation and revealed an average width, thickness, and diameter of 38.29 mm, 14.36 mm, and 112.64 mm, respectively. Important relationships were observed between the divisions of the main tendons and the medial collateral ligament, the posterior side of the knee and popliteus muscle. This information can help to explain knee pathologies and facilitate rehabilitation after surgery.

## 1. Introduction

The pes anserinus is formed by the insertions of the sartorius muscle, the gracilis muscle, and the semitendinosus muscle at the medial side of the knee [1,2,3]. This lies superficial to the insertion of the distal tendon of the semimembranosus muscle (SMBM), which inserts as several tendons to form the deep pes anserinus [1]. There are three tendons in this distal attachment: the direct tendon (DT), the anterior or reflect tendons (AT), and the recurrent tendon (RT), which is also known as oblique popliteal ligament (OPL) [1]. 

These distal tendons insert in different anatomical structures [4,5,6,7,8,9,10,11,12] and have different histological [12,13] and embryological [14,15] characteristics affording them different roles in the stabilization [4,9,11,16,17,18] and proprioception [5,13,19] of the knee joint. However, each tendon will respond differently to traction force, which can produce differing repercussions [7,20,21], including avulsion [20,22], tendinopathy [21], bursitis [14,23], meniscal injury [7,22,24], and capsule tear [20,22]. 

Magnetic resonance imaging is typically preferred for identifying the tendon, its insertions, and any pathology [4,6,25,26], but ultrasound is also considered a good tool [16,17,21,27], especially for specific injuries of the distal semimembranosus muscle [17,21]. 

The utility of ultrasound in this setting requires detailed anatomical knowledge of the muscular fibers of the semimembranosus muscle in relation to the distal tendon and the insertions of that muscle. Improved knowledge will help clinicians to better understand these ultrasound images, posteromedial knee injuries, and post-surgery rehabilitation. 

## 2. Materials and Methods

### 2.1. Study Design and Samples

This observational study involved the ultrasound, anatomical, and histological examination of 38 knees (18 left and 20 right) obtained from the donors of 18 women (47.37%) and 20 men (52.63%), ranging in age from 61 to 92 years old. In addition, four limbs were obtained from fetuses aged 29–38 weeks old for embryology study. 

All limbs had been cryopreserved at −20 °C in the dissection room. The body donor program of the Faculty of Medicine and Health Sciences (Bellvitge Campus) provided all of the specimens. None of the anatomical samples had evidence of traumatic injury of surgical scars. Samples that presented interventions, prostheses, or surgical material were excluded. The study was approved by the local ethics committee (IRB00003099).

Before the study, we injected the femoral artery with black latex (Latex Compound Española S.A., Sabadell, Spain) to facilitate its localization in the ultrasound and anatomical studies. The study then proceeded, in order, through ultrasound, anatomical, and histological examination. 

### 2.2. Ultrasound Study 

Ultrasound was performed with the LOGIQ P9 ultrasound system (GE Ultrasound Korea LTD, Seongnam, Korea) using a high-frequency linear probe (6–15 MHz). The posterior thigh muscles were identified in the transverse and longitudinal axis before examination. The semimembranosus muscle was then visualized 20 cm before it formed the main tendon and was followed distally until the tendon divided on the DT, AT, and RT. 

To corroborate and verify the ultrasound images of the AT, RT with posterior dissection study, an ultrasound-guided injection was made. An ultrasound-guided injection of 2 mL of green, red, or blue dye (Tempera mate arts Titan, Manufactures Titan arts SLU, Sant Fruitós del Bages, Barcelona, Spain) and 1.5 mL of saline with a 20-gage needle was performed at the short axis of the lower extremity to obtain a longitudinal image of the RT or oblique popliteal ligament and at the long axis of the medial side of the knee to visualize the medial collateral ligament. This helped to find the insertion point of the AT and DT at the medial tibial condyle inferior to the medial meniscus. 

### 2.3. Anatomical Study

Anatomical samples were analyzed macroscopically by dissections and anatomical sections after the ultrasound study.

#### 2.3.1. Dissection Procedure

The gross anatomical study was performed by dissecting 34 limbs to observe the injected dye and any anatomical relationships between structures. The dissection was performed using a classical method along the planes of division in the skin, subcutaneous tissue, or adipose tissue to expose the muscular fascia, fascia lata, and crural fascia of the anterior compartment. First, we made a longitudinal lateral incision in the posterior side and two horizontal incisions 40 cm superior and inferior to the knee. The anterior side was dissected in the same way. 

The muscular fasciae were then cut; the sartorius, gracilis, and semitendinosus muscles were identified and removed from proximal to distal; the semimembranosus muscle was identified, and the different tendons and attachments were identified. When dissecting the semimembranosus muscle and its tendon we carried out a manual traction of the main tendon. The location of the dye was studied to confirm the correct injection.

Anthropometric measurements of the width, thickness, and diameter of the semimembranosus muscle were taken with a digital caliper (Mytutoyo ABSOLUTE Solar Caliper Series 500 with ABSOLUTE technology, Coral Spring, FL (USA)) at 10 cm from the medial condyle of the femur and its distal tendon, before the division in the DT, AT, and RT. At each step, images were taken to record our findings (Canon EOS 60D, Ota, Tokyo (Japan)).

The eight fetal knees were dissected using the same technique. 

#### 2.3.2. Sectional Study

A sectional anatomic study was undertaken in six limbs by performing transverse cuts from above the patella to below the tibial tuberosity.

### 2.4. Histological Study

In 5 randomly chosen specimens, we collected samples of comparable size (2 cm × 2 cm) from the AT, DT, and RT. The samples were fixed in 4% formaldehyde processed into paraffin blocks and cut into 4-µm sections before being dyed with a hematoxylin-eosin stain. Fascial thicknesses were measured with a Leica Digital Microimaging Device (Leica DMD108 microscope, Leica Microsystems, Wetzlar, Germany). 

### 2.5. Statistical Analysis

Statistical analysis was performed on all the data obtained for the control variables (i.e., sex, limb side, and age) and the anthropometric variables. The qualitative variables are presented as absolute and relative frequency; quantitative variables are in the median and interquartile range (IQR) and mean with standard deviation. The Shapiro–Wilk test is used to study the existence of normal distribution in quantitative variables. The relationship between categorical and quantitative variables is carried out through Student’s *t*-test (normal distribution) or Mann–Whitney U-test (non-normal). The relationship between the quantitative variables is carried out through the Pearson (normal distribution) or Spearman (non-normal) correlation coefficient.

## 3. Results

### 3.1. Ultrasound Study 

**Figure 1:**
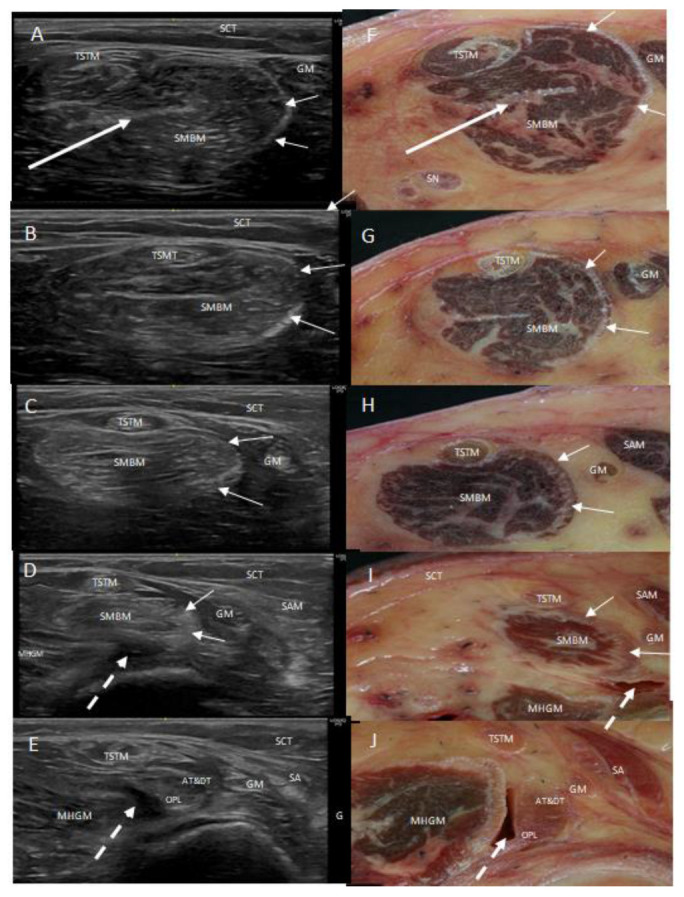
Consecutives ultrasound images (**A**–**E**) and transversal anatomical sections (**F**–**J**) of the posteromedial side of the left thigh. The semimembranosus muscle (SMBM) is visible. The long white arrow shows the intraponeurosis inside the muscle and the short white arrows places the medial aponeurosis that increases from superior to inferior until it arrives to the main tendon and forms the anterior (AT) and direct (DT) tendons of the semimembranosus muscle from the medial to the posterior part of the knee. The oblique popliteal ligament (OPL) comes from the muscle, and it is located at the lateral side. The tendon of the semitendinosus muscle (TSTM) is always in relation to the SMBM. In pictures (**D**,**E**,**I**,**J**), the white discontinued arrow shows the bursa situated between the oblique popliteal ligament and the medial head of the gastrocnemius muscle.

Ultrasound always identified the semimembranosus muscle in the posterior thigh compartment, as an oval or round hypoechogenic structure, with the prove at the short axis, formed by different fascicles with an hyperechogenic line seen in the middle of the muscle (Figure 1A,B).

When the probe followed the muscle inferiorly, a hyperechogenic line appeared at the medial and posterior border of the muscle. This increased distally and followed as a part of the distal tendon. Following the course of the muscle and tendon inferiorly, we observed that this border and muscle rotated from medial to posterior: the medial side of the muscle, with this hyperechogenic border became posterior by the inferior part of the tendon and gave rise to the AT and DT when the tendon divided. The anterior side of the muscle followed the anterior side of the tendon and became lateral and posterior, giving rise to the RT (or oblique popliteal ligament OPL) (Figure 1A–E).

**Figure 2:**
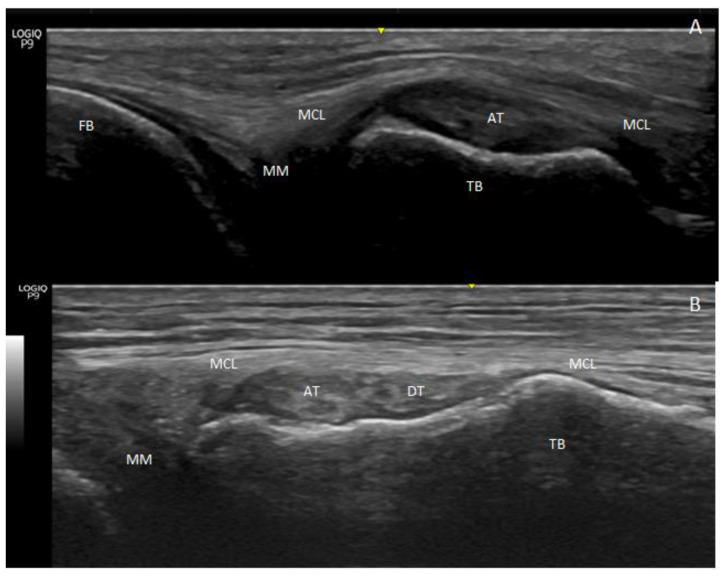
Image of the medial side of the knee. (**A**) An ultrasound image with the prove at the long axis: the anterior tendon (AT) of the semimembranosus muscle is seen as a round hyperechogenic image covered by the medial collateral ligament (MCL) in a special concavity that the tibia bone (TB) forms at the point of insertion. (**B**) Ultrasound vision of the two round hyperechogenic structures compatibles with the anterior tendon (AT) and direct tendon (DT) of the semimembranosus. Medial meniscus—(MM) and femur bone—(FB).

After division, we observed the AT and DT at the long axis as the hyperechogenic structures went to the medial side of the knee. They appeared as round hyperechogenic or hypoechogenic structures, depending on the prove ultrasound position and were covered by the medial collateral ligament that surrounded them and formed a tunnel through which they passed (Figure 2A,B).

**Figure 3:**
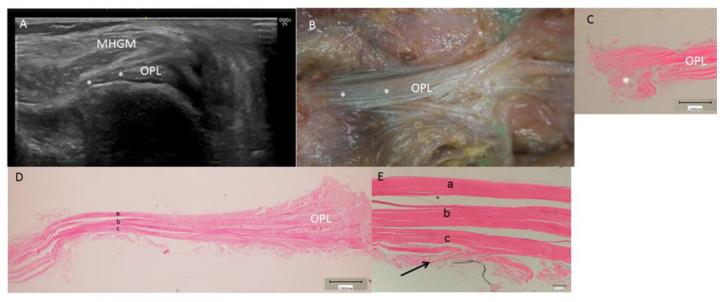
Left oblique popliteal ligament (OPL): (**A**) An ultrasound view at the short axis. The ligament, covered by the medial head of the gastrocnemius muscle (MHGM), inserts posterolateral at the capsule of the knee (*). (**B**) Posterior anatomic view of the left oblique popliteal ligament (OPL) and its insertion at the capsule (*). (**C**) Transversal histologic view of the OPL at the insertion of the capsule (*). (**D**) Transversal histologic view of the OPL. It is possible to visualize several layers of the dense connective tissue (a, b, c) separated by a less connective tissue. (**E**) Magnified vision of the OPL with the three layers (a, b, c) and the deep relation with the capsule of the knee (black arrow).

With the probe in the short axis, the oblique popliteal ligament was observed and located as a long hyperechogenic structure with parallel fibers in close relation to the posterior capsule of the knee, anterior to the medial head of the gastrocnemius muscle (Figure 3A).

**Figure 4:**
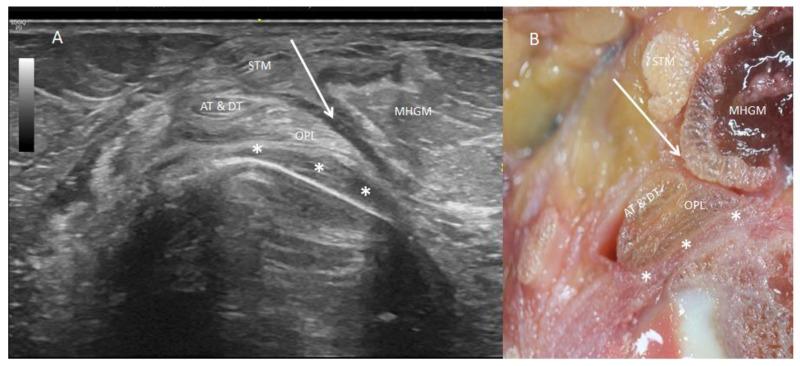
Posterior side of the right knee. (**A**) An ultrasound vision with the prove at the short axis that shows the division of the semimembranosus tendon and the rotation of it. The oblique popliteal ligament (OPL) is seen as a long hyperechogenic structure with parallel fibers in close relation to the posterior capsule of the knee (white *) in a lateral position. The anterior and direct tendon (AT and DT) are in the posterior side. Between these different tendons and the medial head of the gastrocnemius muscle (MHGM) there is the bursa (white arrow). (**B**) A transversal anatomic section of the knee that shows these different structures. Semitendinosus tendon—(STM).

Lateral to the main semimembranosus tendon, between the main semimembranosus tendon and the medial head of the gastrocnemius muscle, all knees revealed a comma-shape hypoechogenic image compatible with a bursa. The presence of a medial hyperechogenic border was a characteristic finding of the medial head of the gastrocnemius muscle (Figure 1D,E and Figure 4A). 

### 3.2. Anatomical Study

**Figure 5:**
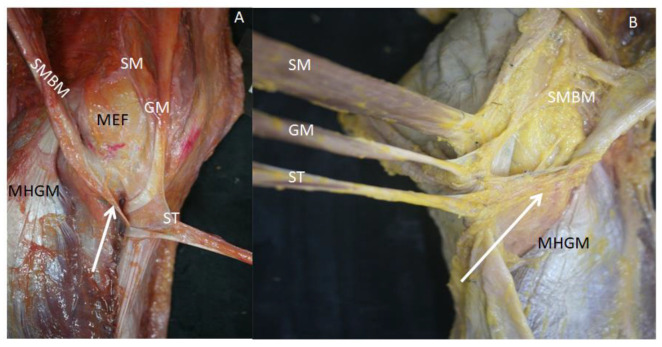
Two posterior and medial vision of a (**A**) left knee and (**B**) right knee. The loose and dense connective tissue expansions (white arrow) go from the pes anserinus (sartorius muscle (SM), gracilis (GM), semitendinosus (ST)) to the tendon of the semimembranosus muscle (SMBM). Medial epicondyle of the femur—(MEF); medial head of the gastrocnemius muscle—(MHGM).

The anatomical study of the knees revealed that the semimembranosus muscle arrived at the medial knee covered by the pes anserinus. After removing each of the tendons that formed the pes anserinus, from proximal to distal, all the knees had lax and dense connective tissue expansions between the pes anserinus and semimembranosus tendons (Figure 5).

**Figure 6:**
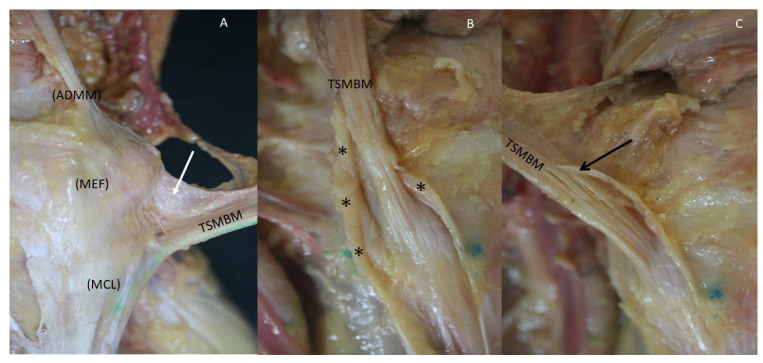
Different visions of the distal tendon of the right semimembranosus tendon (TSMBM) (**A**) Anteromedial vision. Dense connective expansion (white arrow) arrives from the medial collateral ligament (MCL), the medial epicondyle of the femur bone (MEF) and the adductor maximus muscle (ADMM) (**B**) Medial vision. The different expansions (black *) form a tunnel for the TSMBM that merged with it superiorly and it is necessary to cut it to visualize the tendon. (**C**) Posteromedial vision. The tissue that formed the tunnel merged with the tendon (black arrow).

The tendon was also surrounded by connective tissue expansions from the vastus medialis muscle, the fascia lata, the retinaculum, the adductor maximus muscle, the medial collateral ligament, the medial epicondyle, the medial head of the gastrocnemius muscle and popliteus muscle and forming a tunnel for the central tendon that merged with it superiorly. It was necessary to cut this tissue to observe the main tendon (Figure 6). 

**Figure 7:**
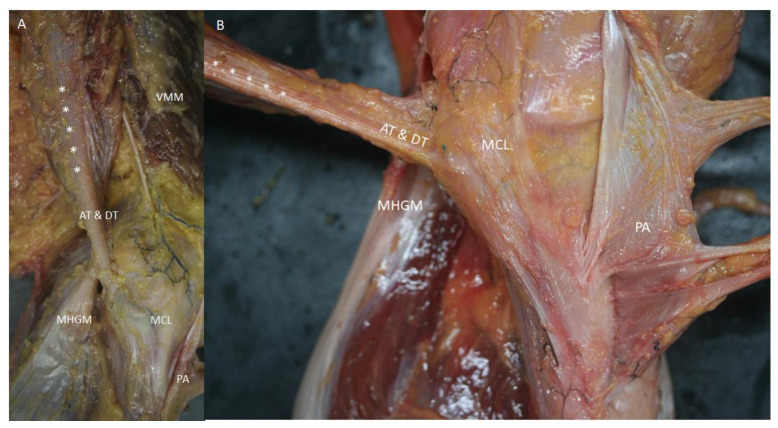
(**A**,**B**) Vision of the medial side of left knee with the pes anserinus (PA) retired. The aponeurosis of the semimembranosus muscle (white *), originated at the medial side of the muscle, follows as the tendon of the anterior and direct tendon (AT and DT) to insert at the medial side of the tibia covered by the medial collateral ligament of the knee—(MCL). Vastus medialis—(VMM); Medial head of the gastrocnemius muscle—(MHGM).

Semimembranosus muscle cuts showed the muscular fibers that formed several fascicles identified in the ultrasound study, and an aponeurosis inside the muscle that corresponded to the end of the proximal aponeurosis of the muscle. At the medial-posterior border, the muscle presented an aponeurotic border that thickened distally (Figure 7). This medial border became more posterior before the tendon divided, forming the DT and AT of the semimembranosus muscle after dividing meanwhile, the anterior part of the muscle and tendon gave rise to the RT (or the oblique popliteal ligament). This converged on the muscle and tendon, confirming the tendon rotation seen on ultrasound (Figure 1F–J). 

**Figure 8:**
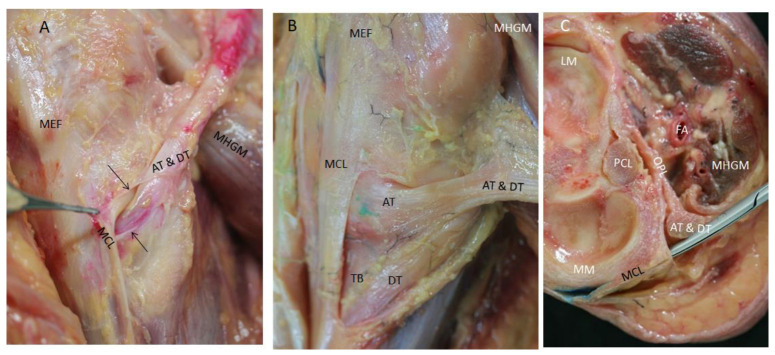
View of the medial side of the right knee (**A**) It is possible to observe the anterior tendon (AT) injected in red by ultrasound-guided injection and direct tendon (DT) before they separate and their relation with the medial collateral ligament (MCL) of the knee. The ligament made a tunnel for both portions (short black arrows). Medial epicondyle of the femur bone (MEF), medial head of the gastrocnemius muscle (MHGM). (**B**) Cutting part of the medial collateral ligament allowed us to visualize the anterior and direct tendons (AT and DT) of the semimembranosus inserted at the medial side of the tibia bone (TB). (**C**) Transversal cut of a right knee allowed us to visualize the disposition of the medial collateral ligament of the knee and its relation with the tendons of the semimembranosus and medial meniscus—(MM). OPL inserts at the posterior side of the capsule of the knee. Lateral meniscus—(LM); Posterior cruciate ligament—(PCL); Medial head of the gastrocnemius muscle—(MHGM); Femoral artery—(FA).

To observe the divisions of the main tendon of the semimembranosus muscle, we needed to remove the medial head of the gastrocnemius muscle from distal to proximal and cut the soleus muscle. We also needed to cut the inferior part of the medial collateral ligament because it made a tunnel and helped to fix the AT and DT, preventing visualization. The dye that marked the AT and DT remained inside this tunnel in all the cases, and even progressed up to the main tendon without extraversion. After cutting the medial collateral ligament, we observed that the AT had a transverse direction and inserted in a fosse of the medial border of the medial condyle of the tibia. The DT followed inferiorly and longitudinally under the medial collateral ligament (Figure 8A,B). At this point, a bursa was located with an inverted U-shape that involved the indirect tendon. 

The RT or oblique popliteal ligament were covered by the medial head of the gastrocnemius muscle but were separated from it by a fascia. After removing the medial head of the gastrocnemius muscle, the entire oblique popliteal ligament had a transverse direction and a superolateral reflection reject to the posterior knee. Its trajectory was from medial to lateral and from inferior to superior, reaching the posterior knee capsule and the lateral condyle of the femur (Figure 3B). Here, there was a deep connection between the tendon and the posterior knee capsule, which were impossible to isolate. An expansion to the meniscofemoral ligament was also observed, but we could not prove any connection between this part of the oblique popliteal ligament and the medial or lateral meniscus. Handmade traction of the main tendon of the semimembranosus muscle did, however, tense the posterior capsule and indirectly mobilize the medial and lateral meniscus. Also, the histological study showed that the oblique popliteal ligament had three layers that were joined by loose connective tissue (Figure 3C,D). Vessels were observed parallel to these layers, and in one case, an arterial vessel crossed the oblique popliteal ligament. 

The dye injected by ultrasound was present at the AT and DT in all cases, confirming the correct visualization by ultrasound, but was only present at the RT in 80% despite this; the dye was always located inferior to the tendon.

Measurements were only recorded in 32 of the 38 knees, because 6 knees were used for transversal cuts. Among these, 18 were from men (56.3%) and 14 were from women (43.8%), while 21 were right-sided (65.6%) and 11 were left-sided (34.4%). The width, thickness, and diameter measurements of the muscle were 38.29 mm, 14.36 mm, and 112.64 mm, respectively; the corresponding measurements for the tendon (before its division) were 11.81 mm, 4.69 mm, and 38.44 mm (Table 1). Statistical analysis showed that neither gender nor laterality affected these measures. Similarly, there were no differences between the muscle and the tendon measurements (Table 2).

No statistically significant differences were observed in the continuous variables that reflect the dimensions of the tendon and muscle. The median and distribution within the variables were the same for each of these continuous variables.

There is no correlation between the width, thickness, or diameter of the tendon and muscle.

All cases had a synovial bursa between the semimembranosus muscle and the medial head of the gastrocnemius muscle before the semimembranosus tendon divided. This corresponded to the hypoechogenic comma-shaped image on the ultrasound. We did not observe the continuity of the capsule of the knee or the tendon of the medial head of the gastrocnemius muscle under the capsule (Figure 1I,J and Figure 4B). 

One specimen showed a variation in its muscular belly width by 0.5 mm before transitioning to the main tendon. This small muscle was separated from the tendon and inserted into the thigh fascia at this point.

**Figure 9:**
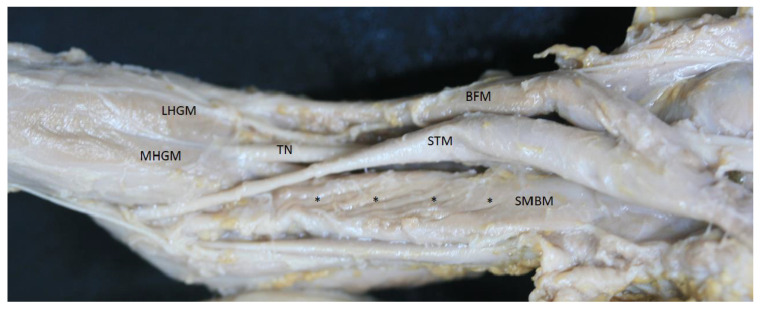
The posterior side of the thigh from fetuses aged 33 weeks old. It shows the posterior muscular fibers (black *) of the semimembranosus muscle (SMBM) without aponeurosis. Semitendinosus—(STM); biceps femoris muscle—(BFM); medial head—(MHGM); lateral head—(LHGM) of gastrocnemius muscle; tibial nerve—(TN).

The embryonic study at different months showed that the anatomy was very similar to that observed in adults. However, we did not observe a distal rotation of the tendon, only seeing posterior muscular fibers and the anteromedial aponeurosis at the side of the muscle (Figure 9). Thereafter, the tendon divided as for adults. 

### 3.3. Histological Study

**Figure 10:**
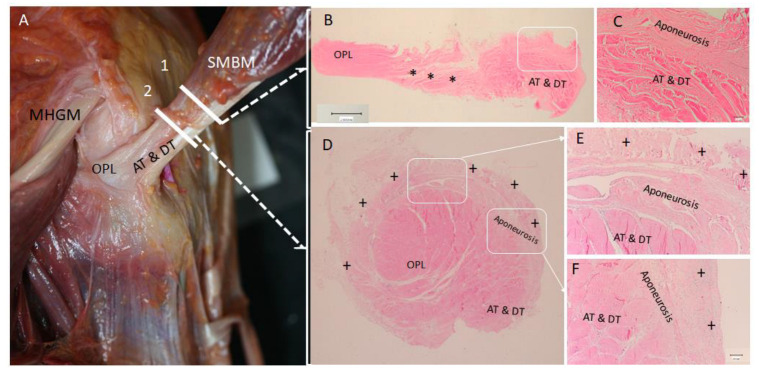
(**A**) An anatomical image of the posteromedial view of a left knee. The semimembranosus muscle (SMBM) will give the anterior and direct tendons (AT and DT) and oblique popliteal ligament (OPL). The medial head of the gastrocnemius muscle (MHGM) is superiorly to OPL. Lines 1 and 2 marked the levels of cut of the semimembranosus muscle. (**B**) Level 1: Transversal and histological view where two different points of the dense connective tissue belong to the anterior (AT) and direct tendons (DT) and oblique popliteal ligament (OPL). They are separated by loose connective tissue (black *). (**C**) It corresponds to a magnified vision of the white square of the picture (**B**). The aponeurosis of the muscle semimembranosus has a deep relation with the AT and DT. It does not surround the OPL. (**D**) It corresponds to a transversal and histological view of the tendon at the level 2, inferiorly just before the different tendons of the SMBM separate. The aponeurosis surrounds the AT and DT but not the OPL. There is other dense connective tissue (black +) that surrounds all the tendons that correspond to the different tissues as the medial collateral ligament, medial epicondyle, popliteal muscle. (**E**) Image of the aponeurosis and the close relation with the AT and DT. (**E**,**F**) corresponds to a magnified vision of the white squares of the pictures.

The histology of the common tendon before it separated into the three main portions showed a difference. RT was notably separated from the AT and DT, which remained together by less dense connective tissue (Figure 10B). Particularly, RT presented tendinous fibers that ran parallel and compact while the AT and DT were also formed by dense connective tissue that ran in several directions and were surrounded by a layer of dense connective tissue, corresponding to the aponeurosis of the medial border of the semimembranosus muscle that became posterior (Figure 10B–E). 

Detailed study of the oblique popliteal ligament showed that it was formed by three layers (as in the anatomical study) separated by lax connective tissue. Also, the anterior layer was related to the capsule and shared connections (Figure 3C,D).

## 4. Discussion

A notable discovery of this study is the helicoidal disposition of the distal semimembranosus muscular and myotendinous fibers before their division into the AT, DT, and RT. We hypothesize that this disposition at its distal insertion affords a better tolerance to traction forces and could protect against potential damage caused by injury. This is similar to the proximal insertion of the semimembranosus muscle at the ischial tuberosity, where it is inserted with a double tendinous portion and oriented in several angles. As a protective factor, this could account for the lower incidence of injuries when compared to the semitendinosus muscle and the long head of the biceps femoral tendon [28]. The distal insertion with rotation is secured for different expansions to anatomical structures, as described in the results, that fix the main tendon and maintain its rotation. Although we cannot compare this description to previous studies, other authors have described these expansions as part of the different insertions of the main tendon [11]. 

Regarding the insertion of some of the RT or oblique popliteal ligament fibers at the meniscus, we could not show direct insertion at the lateral meniscus [4,10] or medial meniscus, as described elsewhere [29]. However, the close association of the oblique popliteal ligament with the knee capsule could explain the capsular tears and meniscus injuries [12]. We observed fibers of the external layer of the knee capsule surrounding the semimembranosus tendon, demonstrating continuity of the joint capsule with the posterior horn of the medial meniscus. Results also showed that applying a traction force to the semimembranosus tendon had repercussions in the posterior knee capsule and medial meniscus. This shows the importance of the oblique popliteal ligament in the knee capsule and the need to consider it in rehabilitation treatments involving these structures. Other authors consider this a possibility and contraindicate active work on the semimembranosus muscle in early rehabilitation due to the possible negative impact of traction of the semimembranosus distal tendon on sutures of the posterior horn of the medial meniscus, through the posterior joint capsule [29,30,31,32]. No consensus has been reached [30,32,33].

Our study cannot verify whether active work on the semimembranosus muscle can benefit or harm the initial rehabilitation of such interventions. It does, however, allow us to argue that any semimembranosus action that involves oblique popliteal ligament traction at this site will have repercussions on the joint capsule of the knee and on the medial meniscus (i.e., whether active or passive; in an open, closed or mixed kinetic chain; in internal, external, total, or intermediate working amplitude; or in an isometric, concentric or eccentric way). Therefore, this requires consideration in the initial phase of post-surgical rehabilitation for repairs to the posterior horn of the medial meniscus. Early, low-intensity traction may be beneficial by facilitating proprioceptive awakening, whereas early high-intensity traction can be harmful due to the risk of tearing an unhealed suture.

These relationships between the capsule and the medial meniscus and the impact or semimembranosus muscle traction on both could also explain why the distal insertions of the semimembranosus muscle follow different tendons. If it was only anchored to one in the posterior part of the joint capsule, excessive forces could cause injury through avulsion of the anatomical structures on which it exerted traction. This also relates to the observed origin of the oblique popliteal ligament which arises directly from the muscular part of the semimembranosus muscle. By contrast, the architecture with multiple insertions that form the deep pes anserinus allows the total force exerted by the semimembranosus muscle to be distributed between the AT, DT, and RT when flexing (in an open kinetic chain), or stabilizing (in a closed kinetic chain) the knee, breaking the force down into different vectors will minimize the risk of traction injury.

The origin of the DT and AT from the medial aponeurosis, which becomes the principal component of the tendon, could also explain the different mechanisms of injury to the medial knee and medial collateral ligament. However, this would be countered by the many connections of medial anatomical structures that we have described.

The differences in disposition between the fetal and adult muscles is an important finding. Although the insertion points of the distal tendons of the semimembranosus muscle change during gestation and although its final disposition may be modified even after birth [15] it was notable that the rotation of the muscle and tendon was only present in adults. The lack of this rotational component in the different fetal periods may reflect the absence of certain mechanical stimuli while in uterus, such as traction stimuli on the tendon or load stimuli on the lower extremities. It is known that these trigger adaptations and structural changes in the myotendinous tissues on which they act [34,35]. Thus, unlike simple movement stimuli that are present from 7–8 weeks of gestation [36], the semimembranosus muscle does not exert or receive biomechanical stresses that fight against gravity and does not have need to exert a stabilizing action under loading on the knee or on the lumbopelvic complex. This makes the helical disposition unnecessary until after birth, when the muscle will be subject to the demands of standing and the need for a stabilizing action. Such a change in stressors probably stimulates the adult arrangement of distal insertions with anchoring in different tendons to allow greater resistance to traction and a better distribution of the forces exerted by muscle contraction which is facilitated by the rotational component.

This study has some limitations that should be considered, as the total height of the donors and the body mass index that are not included in the study. Nevertheless, these parameters do not modify the morphology of the analyzed structures and the helical arrangement of the semimembranosus muscle and tendon. Future studies should confirm these results considering the proposed limitations.

## 5. Conclusions

The results of this study showed that ultrasound is a valid tool for identifying the divisions of the distal tendon of the semimembranosus muscle and its relationship with the adjacent anatomical structures.

Anatomically, it has been proven that the distal fibers of the semimembranosus muscle adopt a helical arrangement that continues and extends until its division into the three main distal tendons. This helical arrangement does not appear in fetuses between 29 and 38 weeks of age.

No direct anatomical continuity has been observed between the oblique popliteal ligament and the meniscus, but it has been proven that applying a traction force on the tendon of the semimembranosus muscle has an indirect impact on the medial meniscus due to the transmission of force traction through the posterior capsule of the knee. This point should be considered in the early stages of rehabilitation of a posterior horn of the medial meniscus repair.

## Figures and Tables

**Table 1:** Morphometry of the tendon and muscle semimembranosus 95% IC: 95% confidence interval. SD—Standard deviation.

	TENDON (mm)			MUSCLE (mm)		
	Width	Thickness	Diameter	Width	Thickness	Diameter
Average (SD)	11.8 (1.43)	4.69 (0.72)	38.44 (2.93)	38.29 (4.33)	14.36 (3.23)	112.64 (10.74)
95% CI	11.30–12.32	4.43–4.95	37.38–39.50	36.73–39.85	14.36–13.2	108.77–116.51
Median (IQR)	12.21 (2)	4.65 (0.96)	38.77 (2.5)	39 (5.08)	13.1 (4.43)	109.8 (22.42)
Test Shapiro–Wilk (*p*)	0.86 (0.000)	0.961 (0.20)	0.879 (0.14)	0.940 (0.07)	0.85 (0.001)	0.894 (0.004)

**Table 2:** Morphometry of the tendon and muscle (10 cm before the tendon) semimembranosus according to the sex and side.

Measures Distribution of Tendon and Muscle According to the Sex ^(1)^		SEX		Test U Mann–Whitney
		Man	Woman	
TENDON (mm)	width	11.70 (1.48) 10.96–12.3	11.96 (1.39) 11.15–12.76	140.5 (0.582)
	thickness	4.91 (0.59) 4.61–5.20	4.41 (0.78) 3.95–4.86	80.0 (0.081)
	diameter	38.41 (3.28) 36.78–40.0	38.47 (2.53) 37–39.9	130.0 (0.879)
MUSCLE (mm)	width	38.5 (4.23) 36.4–36.44	37.9 (4.59) 12.38–15.1	101.0 (0.342)
	thickness	14.85 (2.35) 12.97–16.7	13.74 (2.33) 12.38–15.1	110.0 (0.558)
	diameter	111.2 (10.1) 106.2–116.2	114.5 (11.7) 107.8–121.3	147.0 (0.425)
Measures distribution of tendon and muscle according to the side ^(1)^		SIDE		
		Right	Left	
TENDON (mm)	width	11.83 (1.34) 11.23–12.44	11.76 (1.65) 10.66–12.87	126.0 (0.696)
	thickness	4.69 (0.68) 4.39–501	4.67 (0.82) 4.13–5.23	117.5 (0.938)
	diameter	38.57 (3.12) 37.15–39.99	38.19 (2.66) 36.40–39.97	103.0 (0.639)
MUSCLE (mm)	width	37.85 (4.52) 35.80–39.91	39.12 (3.99) 36.44–41.81	129.0 (0.611)
	thick	14.49 (3.59) 12.85–16.12	14.13 (2.54) 12.42–15.83	115.0 (1.0)
	diameter	111.38 (10.87) 107.96–122.16	115.1 (10.57) 107.96–122.16	145.0 (0.254)

^(1)^ Average, standard deviation, 95% confidence interval of the average.

## Data Availability

The datasets used in this work can be accessed upon request.
